# Effects of Transcranial Direct Current Stimulation on the Recognition of Bodily Emotions from Point-Light Displays

**DOI:** 10.3389/fnhum.2015.00438

**Published:** 2015-08-03

**Authors:** Sharona Vonck, Stephan Patrick Swinnen, Nicole Wenderoth, Kaat Alaerts

**Affiliations:** ^1^Movement Control and Neuroplasticity Research Group, Department of Kinesiology, KU Leuven, Leuven, Belgium; ^2^Faculty of Medicine and Life Sciences, Hasselt University, Hasselt, Belgium; ^3^Department of Health Sciences and Technology, ETH Zurich, Zurich, Switzerland

**Keywords:** transcranial direct current stimulation, superior temporal sulcus, emotion recognition, point-light displays, autism, neuromodulation

## Abstract

Perceiving human motion, recognizing actions, and interpreting emotional body language are tasks we perform daily and which are supported by a network of brain areas including the human posterior superior temporal sulcus (pSTS). Here, we applied transcranial direct current stimulation (tDCS) with anodal (excitatory) or cathodal (inhibitory) electrodes mounted over right pSTS (target) and orbito-frontal cortex (reference) while healthy participants performed a bodily emotion recognition task using biological motion point-light displays (PLDs). Performance (accuracy and reaction times) was also assessed on a control task which was matched to the emotion recognition task in terms of cognitive and motor demands. Each subject participated in two experimental sessions, receiving either anodal or cathodal stimulation, which were separated by one week to avoid residual effects of previous stimulations. Overall, tDCS brain stimulation did not affect the recognition of emotional states from PLDs. However, when emotions with a negative or positive–neutral emotional valence were analyzed separately, effects of stimulation were shown for recognizing emotions with a negative emotional valence (sadness and anger), indicating increased recognition performance when receiving anodal (excitatory) stimulation compared to cathodal (inhibitory) stimulation over pSTS. No stimulation effects were shown for the recognition of emotions with positive–neutral emotional valences. These findings extend previous studies showing structure–function relationships between STS and biological motion processing from PLDs and provide indications that stimulation effects may be modulated by the emotional valence of the stimuli.

## Introduction

The human ability to perceive and understand others actions, emotions, and intentions is pivotal to the formation of social interactions, communication, and relationships. At the neural level, studies have revealed a set of brain regions which are part of the action perception network or mirror system and which are particularly involved in human motion perception (Gallese et al., 2004; Rizzolatti and Craighero, 2004; Rizzolatti and Fabbri-Destro, 2008). The existence of “mirror” neurons was first described in the macaque monkey brain using single-cell recordings (di Pellegrino et al., 1992). Later, neuroimaging and neurophysiological research confirmed the existence of a similar “fronto-parietal” mirror system in the human brain (Fadiga et al., 1995; Grafton et al., 1996; Iacoboni et al., 1999). In addition to the mirror-motor areas in the inferior parietal, inferior frontal gyrus and premotor cortex, the action perception network also encompasses higher-order visual areas in the posterior superior temporal sulcus (pSTS) that convey visual input to downstream visuo-motor areas (Rizzolatti and Craighero, 2004; Caspers et al., 2010; Molenberghs et al., 2010, 2012). Especially the *right* pSTS has been highlighted as a key neural locus for perception of bodies and biological motion (Allison et al., 2000), as it has been shown to be particularly activated during the perception of eye or mouth movements, body language, and biological motion (Allison et al., 2000; Hein and Knight, 2008; Redcay, 2008; Carrington and Bailey, 2009).

Prior research most frequently adopted the so-called point-light displays (PLDs) to study the neural basis of biological movement perception as they represent a highly controllable, impoverished form of biological motion (Johansson, 1973). In these displays, only a few bright moving point-light dots are depicted against a dark background to represent the movement of the main joints of a human body. Although they lack any information on texture or form, PLDs are highly salient in depicting human actions as well as information on the gender or emotional state in which the point-light figure is engaged (Johansson, 1973; Cutting and Kozlowski, 1977; Dittrich et al., 1996; Pollick et al., 2002, 2005; Alaerts et al., 2011).

To date, two studies investigated the causal relationship between the ability to perceive PLDs and intact functioning of the (right) pSTS (Grossman et al., 2005; van Kemenade et al., 2012). In these studies, repetitive transcranial magnetic stimulation (rTMS) was used to temporarily disrupt activity in right pSTS and both reported deteriorated performance on a task involving the detection of biological motion in noise-masked displays (Grossman et al., 2005; van Kemenade et al., 2012).

Here, we used a transcranial direct current stimulation (tDCS) paradigm to investigate whether neural information processing can be up- or down-regulated in order to increase or deteriorate biological motion perception. Development of brain stimulation paradigms that can *enhance* biological motion perception abilities would be of particular interest for neuropsychiatric conditions that have clear implications in social and communicative domains, such as autism spectrum disorders (ASD). At the behavioral level, ASD-related deficiencies in biological motion perception have been reported repeatedly (Kaiser and Pelphrey, 2012) and also previous neuroimaging studies from our and other labs showed altered activity and connectivity patterns in pSTS regions in the autistic brain (Freitag et al., 1994; Pelphrey et al., 2011; Alaerts et al., 2014). Particularly tasks including an emotion processing component (e.g., reporting the emotional state of PLD figures) have shown highly consistent ASD-specific deficiencies (Hubert et al., 2007; Parron et al., 2008; Atkinson, 2009; Nackaerts et al., 2012; Alaerts et al., 2014).

In the present study, we applied tDCS with anodal (excitatory) or cathodal (inhibitory) electrodes mounted over right pSTS (target) and orbito-frontal cortex (reference) in healthy human subjects while they performed a bodily emotion recognition task using PLDs. Performance [accuracy and reaction times (RTs)] was also assessed on a control task that was matched to the emotion recognition task in terms of cognitive and motor demands. Each subject participated in two experimental sessions, receiving either anodal or cathodal stimulation, separated by one week to avoid residual effects of previous stimulations. Based on previous studies and in the light of our anatomical hypothesis, we expected anodal (excitatory) stimulation over right pSTS to increase sensitivity to biological motion thereby enhancing emotion recognition abilities. Cathodal (inhibitory) tDCS over pSTS is expected to reduce or have no effect on performance.

## Materials and Methods

### Participants

Twenty-four subjects participated in the study (15 females, 22.35 ± 0.28 years). All participants were naive regarding the purpose of the experiments and were unfamiliar with PLDs. Subjects had no history of medical, neurological, or psychiatric disorders, and none of them were receiving acute or chronic medication affecting the central nervous system.

Written informed consent was obtained from all participants prior to the experiment. Consent forms and study design were approved by the local Ethics Committee for Biomedical Research at the KU Leuven in accordance to the Code of Ethics of the World Medical Association (Declaration of Helsinki).

### General procedure

**Figure 1:**
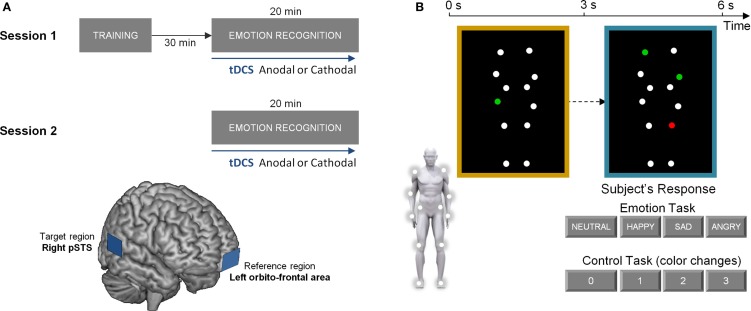
**Experimental protocol and experimental tasks**. **(A)** visualizes the experimental protocol, consisting of two stimulation sessions separated by one week. At each session, participants received either anodal (excitatory) or cathodal (inhibitory) tDCS over the right posterior superior temporal sulcus (pSTS). The lower part of **(A)** represents a schematic presentation of the placement of the anodal/cathodal electrodes over the target (right pSTS) and reference region (left orbito-frontal area). **(B)** visualizes one trial of the experimental tasks. In the *emotion recognition task*, participants were instructed to indicate the emotional state of the blue-bordered PLD relative to the baseline yellow-bordered PLD (always showing a neutral emotional state). The same PLD-stimuli were presented in the *control task* matched for cognitive and motor demands. Here, one of the dots in the yellow-bordered PLD briefly changed color to either red or green. Subsequently, participants had to indicate the number of dots that changed into the same color in the blue-bordered PLD (2 in this example).

All subjects received tDCS during the completion of a bodily emotion recognition task and a control task. They participated in two stimulation sessions where tDCS was applied with anodal (excitatory) or cathodal (inhibitory) electrodes mounted over the right posterior superior temporal sulcus (pSTS) (target) and left orbito-frontal cortex (reference). Sessions were separated by one week to avoid residual effects of previous stimulations (Figure 1A).

Prior to the first stimulation session, subjects were familiarized with task instructions and were allowed to practice the tasks. After a 30-min resting period, tDCS was administered while subjects performed alternating blocks of the emotion recognition task and the control task. Task performance was initiated 5 min after tDCS onset and lasted 8.5 min. TDCS was presented for 20 min to ensure that it was applied during the entire duration of the tasks. Half of the participants received anodal stimulation in the first stimulation session. The other half started with cathodal stimulation (Figure 1A).

### Transcranial direct current stimulation

Transcranial direct current stimulation was delivered by an electrical constant direct current stimulator (HDC Stim, Newronika), which was connected to a pair of rectangular electrodes (25 cm^2^). The stimulating current was an anodal or cathodal constant current of 1.5 mA, delivered for 20 min. In the anodal condition, the anode was placed over right temporal lobe, targeting the posterior part of the STS and the cathode was placed over the left orbito-frontal area (see Figure 1A for a schematic presentation of the target and reference regions). In the cathodal condition, the placement of the two electrodes was reversed. A similar mild itching sensation was felt by the subjects from receiving either anodal or cathodal stimulation such that subjects were blinded on the type of stimulation that was administered. The electrode over pSTS was covered with conducting gel [Zero-gel (ECG–EEG)] to improve conduction through the hair and scalp, the orbito-frontal electrode was covered with a conducting gel and inserted into a saline-soaked synthetic sponge to improve the conduction. Pilot work indicated that this procedure was optimal for ensuring that low impedance between the electrodes and the scalp was maintained over the course of the experiment.

To determine the position of the target electrode over the pSTS region, we used averaged pSTS scalp positions determined in four participants (two of which also participated in the actual study) (age range: 25–30 years; three males; one female) using a Visor neuronavigation system. High-resolution T1-weighted anatomical brain images (Siemens 3T scanner) were collected for these four subjects using a magnetization prepared rapid gradient echo sequence [MPRAGE; repetition time (TR) = 2300 ms, echo time (TE) = 2.98 ms, 1 mm × 1 mm × 1.1 mm voxels, field of view (FOV): 240 × 256, 160 sagittal slices]. Subsequently, the Visor neuronavigation system (Advanced Neuro Technology, the Netherlands) was used to localize the pSTS (defined by MNI coordinates *x* = 53, *y* = −53, *z* = 9) relative to the inion-nasion and the right ear tragus. The MNI coordinates were derived *a priori* and represent average coordinates of 12 studies reporting pSTS-activation during biological motion perception (Jastorff and Orban, 2009) (see Figure 1A for a schematic presentation).

### Emotion recognition from point-light displays

In both of the administered tasks (emotion and control), stimuli consisted of moving PLDs of a male and female actor. Stimuli were based on motion capture data as previously described (Alaerts et al., 2011, 2014; Nackaerts et al., 2012). In short, 12 reflective markers, attached to the joints of the ankles, the knees, the hips, the wrists, the elbows, and the shoulders, were tracked using an eight-camera VICON system (capturing system measuring at 100Hz, Oxford Metrics, Oxford, UK) (Figure 1B). In the adopted movie files (duration 3 s), marker positions were visible as 12 moving white spheres on a black background from three different viewpoints [front view (0°), side view (90°), and intermediate view (45°)]. The moving dots subtended 11 × 12 degrees visual angle at an approximate viewing distance of 50 cm. Each dot subtended 0.25 degrees. The stimuli portrayed human actions (walking; jumping; kicking) that expressed four bodily emotional states: sadness, anger, happiness, or neutral.

In the *emotion recognition task*, each trial showed a yellow-bordered PLD, followed by a blue-bordered PLD (Figure 1B). Participants were asked to indicate as fast and accurate as possible whether the presented point-light figure in the blue-bordered movie moved in a different “emotional state” compared to the point-light figure in the yellow-bordered movie. The emotional state of the blue-bordered PLD could either be indicated as sadder, angrier, happier, or not different (neutral) from the yellow-bordered PLD which always showed a point-light figure in a neutral emotional state.

In the *control task*, participants had to indicate color changes in the point-lights. In the yellow-bordered PLD, one of the dots briefly (0.5 s) changed color to either “red” or “green” at a random time-point. Subsequently, participants had to indicate the number of dots (0–1–2–3) that changed into the same color in the blue-bordered PLD (Figure 1B).

Response options were displayed at the bottom of the screen, which corresponded to four response buttons on a response box. Participants always used the right index, middle, ring, and little finger for button pressing. TRs and accuracy rates were assessed using E-Prime software (Psychological Software Tools).

During practice, participants completed two blocks of the emotion recognition task, alternated with blocks of the control task. During each of the stimulation sessions (anodal/cathodal), subjects performed five blocks of the emotion recognition task, alternated with blocks of the control task. Each block consisted of five trials, such that 25 responses were collected for each task within each testing session. In each trial, the (blue-bordered) PLD expressed one of four bodily emotional states: anger (seven trials), happiness (six trials), sadness (six trials), and neutral (six trials).

### Data analysis and statistics

For each task, the correct RT and percentage correct answers (accuracy) were assessed for each stimulation condition (anodal, cathodal over pSTS), and a single inverse efficiency index (IEI) was calculated by dividing the RT scores by accuracy (RT/accuracy).

Effects of tDCS stimulation on task performance were analyzed by conducting a 2 × 2 repeated measures ANOVA analysis with “stimulation” (anodal, cathodal over pSTS) and “task” (emotion, control) as within-subject factors.

To assess the possibility of learning effects on task performance (irrespective of stimulation type), a similar 2 × 2 repeated measures ANOVA analysis was performed with “session” (session 1, session 2) and “task” (emotion, control) as within-subject factors.

The level of significance was set at a *p*-value of.05.

Statistica 10.0 (StatSoft. Inc., Tulsa, USA) was used for statistical analyses.

## Results

### Effects of tDCS

**Figure 2:**
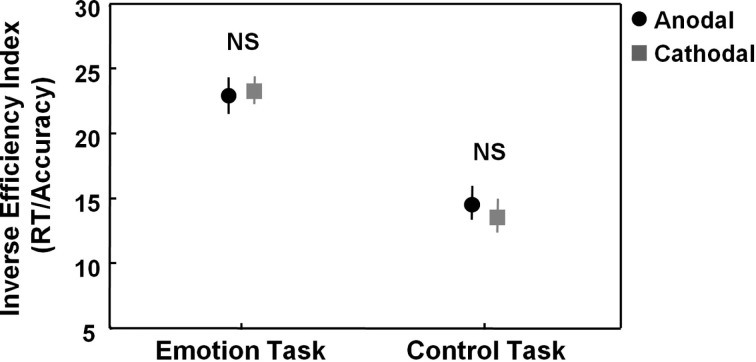
**Effects of tDCS on the emotion and control task**. Figure 2 visualizes task performance [inverse efficiency index (RT/accuracy)] on the emotion and control task while participants received anodal (excitatory) (black circles) or cathodal (inhibitory) tDCS over pSTS (gray squares). Main analyses showed no effects of stimulation on the emotion or control task. Vertical bars denote standard errors. NS, not significant.

The main 2 × 2 repeated ANOVA analysis on the IEI (RT/accuracy) revealed no significant main effect of stimulation [*F*(1, 23) = 0.04, *p* = 0.84] or “task × stimulation” interaction [*F*(1, 23) = 0.93, *p* = 0.35], indicating that tDCS has no overall effect on task performance of the emotion task or control task (Figure 2).

**Figure 3:**
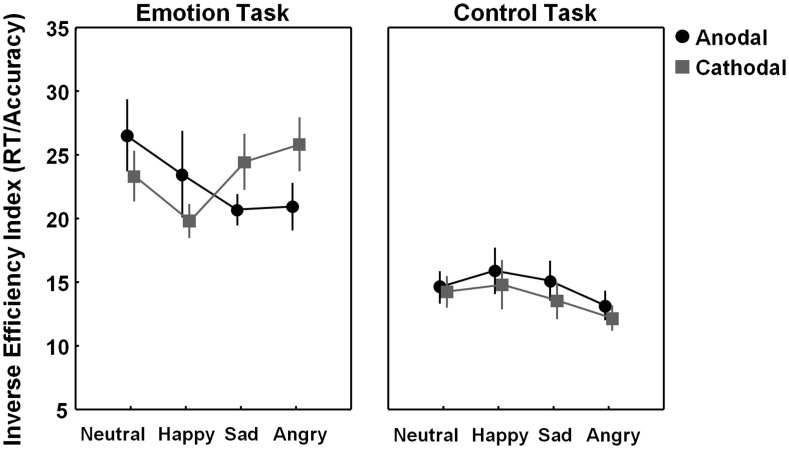
**Visualization of tDCS effects separately for each emotion category (neutral, happy, sad, and angry)**. Figure 3 visualizes task performance [inverse efficiency index (IEI) (RT/accuracy)] on the emotion and control task while participants received anodal (excitatory) (black circles) or cathodal (inhibitory) tDCS over pSTS (gray squares). Exploratory analysis indicated that for the emotion task, not for the control task, effects of stimulation are modulated by emotion type. Vertical bars denote standard errors.

To check for more subtle and specific effects of stimulation, an exploratory 2 × 2 × 4 ANOVA analysis was conducted, additionally including the factor “emotion category” (neutral, happy, sad, angry). The “task × stimulation × emotion category” three-way interaction showed a non-significant trend [F(3, 69) = 2.35, *p* = 0.08], tentatively indicating that stimulation effects modulated differently depending on emotion category and task. Particularly, as visualized in Figure 3 (*left panel*), tDCS tended to exert a differential effect on recognizing the sad and angry emotional states (i.e., emotions with “negative emotional valence”), as compared to recognizing the happy and neutral emotional states. To further explore a possible contribution of “negative emotional valence,” a 2 × 2 × 2 ANOVA analysis was conducted with the factors “task” (emotion, control), “stimulation” (anodal, cathodal over pSTS), and “emotional valence,” directly contrasting “negative emotional valence” stimuli (sad and angry combined) with “non-negative” valence stimuli (happy and neutral combined). This exploratory ANOVA analysis confirmed that effects of stimulation were significantly modulated by emotional valence at least for the emotion task, not for the control task (significant “task × stimulation × emotional valence” three-way interaction [F(1, 23) = 7.67, *p* = 0.01]). Particularly, for recognition of the “negative valence” stimuli (angry–sad), performance was significantly higher (lower IEI) in the pSTS-anodal (excitatory), compared to the pSTS-cathodal (inhibitory) stimulation session [F(1, 23) = 6.62, *p* = 0.03, *p*-value Bonferroni corrected for multiple comparisons], whereas for recognizing emotional states with a “non-negative” positive–neutral emotional valence, no significant effect of stimulation was revealed [F(1, 23) = 2.18, *p* = 0.31, corrected].

Overall, a similar modulation of performance was revealed for the accuracy and RT measures separately (visualized in Figure S1 in Supplementary Material).

### Learning effects

No general learning effects were revealed on the emotion or control task indicating that overall performance (across different emotion types) did not increase from session 1 to session 2 [*F*(1, 23) = 1.66, *p* = 0.21]. Also no “task × session” interaction was revealed [*F*(1, 23) = 1.9, *p* = 0.18]. However, a significant “task × session × emotion type” three-way interaction [*F*(3, 69) = 3.69, *p* = 0.02] indicated that session effects modulated differently depending on emotion type and task.

**Figure 4:**
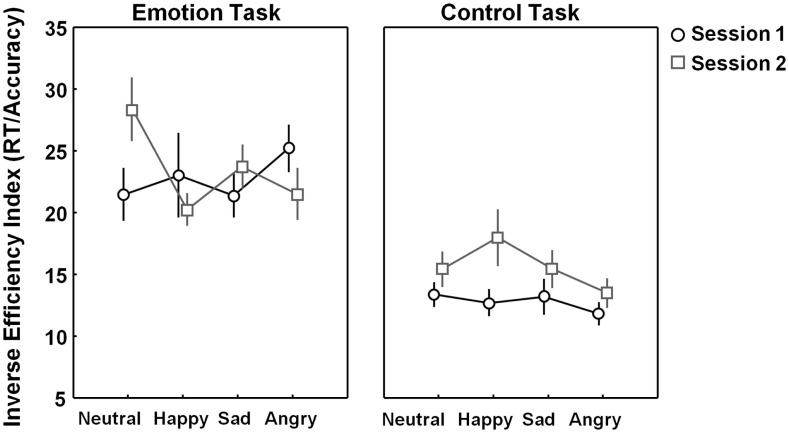
**Learning effects visualized separately for each emotion category (neutral, happy, sad, and angry)**. Figure 4 visualizes task performance [inverse efficiency index (RT/accuracy)] on the emotion and control task on session 1 (black open circles) and session 2 (gray open squares). No significant learning (session-) effects were revealed on the emotion or control task. Vertical bars denote standard errors.

As seen in Figure 4 (*right panel*), performance on the control test tended to be higher in session 1 compared to session 2, but for none of the emotion categories, these differences reached significance after correction for multiple comparisons [all, *p* > 0.05, corrected]. Also for performance on the emotion task, no significant effects of session were revealed for any of the emotion categories [all, *p* > 0.05, corrected] (Figure 4, *left panel*).

## Discussion

In this study, we investigated the role of the posterior superior temporal sulcus (pSTS) in emotion recognition from PLDs. To do so, tDCS was applied using anodal (excitatory) or cathodal (inhibitory) electrodes mounted over right pSTS (target) and orbito-frontal cortex (reference) in two separate sessions. Half of the subjects received anodal stimulation over pSTS on the first session, the other half started with cathodal stimulation over pSTS with at least one week in-between.

Based on our primary analysis, we found no evidence that general emotion recognition from PLDs was affected by receiving anodal or cathodal stimulation over pSTS. However, exploratory analyses provided indications that effects of stimulation were dependent on the emotional valence of the presented stimuli. Particularly, when effects of stimulation were explored separately for emotion categories with a “negative” (sadness–anger) or “positive–neutral” (happy, neutral) emotional valence, it was revealed that performance scores for recognizing emotional states with a negative emotional valence significantly increased from receiving anodal (excitatory) stimulation compared to cathodal (inhibitory) stimulation over pSTS, whereas no stimulation effects were shown for recognizing emotional states with a positive–neutral emotional valence. No effects of stimulation were revealed for performance on the control task. Although effects are preliminary, these results provide first indications that tDCS stimulation over pSTS can modulate (negative) bodily emotion recognition from PLDs.

While anodal/cathodal tDCS was targeted over the pSTS, reference electrodes were placed over left orbito-frontal cortex. Similar supra-orbital reference placements have been adopted previously in studies from our (Zhang et al., 2014) and other (Nitsche and Paulus, 2000) labs targeting primary motor cortex to constrain stimulation effects to frontal areas. In our study, it is likely that tDCS affected excitability in orbito-frontal and prefrontal cortex, thereby modulating excitability in a large fronto-temporal network, potentially overlapping with the mentalizing network or social brain [e.g., Allison et al. (2000)]. In depressed patients, excitability-enhancing tDCS over dorso-lateral prefrontal cortex has been shown to improve performance in an affective go-no-go task for positive emotional content (Boggio et al., 2007). Also in healthy participants, prefrontal tDCS has been shown to diminish emotional valence of unpleasant pictures (Boggio et al., 2009; Maeoka et al., 2012) or subtly improve emotional face identification (Nitsche et al., 2012) [but see Plazier et al. (2012)]. Future experiments are warranted to formally assess whether reference electrode placement to areas other than orbito-frontal sites may qualitatively alter the reported effects. Also other procedures can be adopted to increase focality and stimulation efficacy to the targeted region, such as high-definition tDCS using a 4 × 1 electrode placement (constraining current flow to a circumscribed area of four reference electrodes surrounding the target electrode).

To our knowledge, this is the first study to explore the effects of anodal/cathodal tDCS over the posterior STS. In the past, two studies have applied rTMS over the pSTS region during perception of PLDs. In one study, Grossman et al. (2005) applied rTMS over right pSTS and subsequently measured sensitivity to biological motion. The task required subjects to recognize canonical (upright) point-light animations and 180° inverted animations. Here, rTMS resulted in a decreased sensitivity for recognizing upright biological motions, but stimulation did not affect performances for recognizing the inverted animations (Grossman et al., 2005). In another study by van Kemenade et al. (2012), TMS was applied over the left pSTS, the ventral premotor cortex and the vertex (control site). The task involved the recognition of PLDs of human animations masked in noise from scrambled versions of the same stimuli and performances were measured before and after 20 s of continuous theta burst stimulation of premotor, pSTS, and the control site (each tested on different days). Here, authors reported a significant decrease in detection of human PLDs when disrupting the premotor cortex and a marginally significant decrease in detection when left pSTS was stimulated (van Kemenade et al., 2012).

While Grossman et al. (2005) and van Kemenade et al. (2012) used PLDs to assess effects of rTMS over pSTS, other studies used different task paradigms to assess the role of STS in emotion or motion processing. For example, Pitcher (2014) measured the effects of rTMS over right STS during the recognition of facial emotional expressions and found that repetitive TMS specifically deteriorated performance on the facial emotion recognition task, not on a matched control task requiring the identification of faces. Another study applied rTMS over left STS during action observation, and showed that the “mapping” of observed “implied” actions onto the observer’s motor system (as measured by changes in cortico-motor excitability in primary motor cortex) was enhanced from stimulating left STS (Avenanti et al., 2013).

More recently, Arfeller et al. (2013) used fMRI to explore changes in the functional connections of left and right pSTS after application of rTMS. Interestingly, brain regions showing changes were identified in lateral temporo-occipital cortex, the anterior intraparietal region and in the ventral premotor cortex. Considering that these are all regions of the action perception or “mirror” network, the authors concluded that STS plays a pivotal relay position during the observation of others movements (Arfeller et al., 2013). This notion conforms to findings from an earlier lesion study showing that lesions in superior temporal and premotor areas have causal relationships to deficits in biological motion perception (Saygin, 2007).

In the present study, tDCS stimulation mildly affected emotion recognition from PLDs, at least for emotional states with a “negative” emotional valence. One study similarly showed that tDCS over the cerebellum specifically affected recognition of negative facial emotions (anger, sadness), not of positive or neutral faces (Ferrucci et al., 2012). A selective effect for negative emotional stimuli was also reported in a study by Candidi et al. (2011), showing that rTMS over right pSTS specifically deteriorated the detection of threatening human body postures, not the detection of neutral postures (Candidi et al., 2011). Also repetitive TMS over the frontal sensorimotor system specifically interfered with the recognition of faces expressing anger and fear, not happy or neutral faces (Balconi and Bortolotti, 2012). Several studies on emotional face processes indicated that distinct emotions may be processed differently. For example, by using the “face-in-the-crowd” paradigm, Ohman et al. (2001) showed that among faces expressing various emotions, a face expressing anger was processed faster and more accurately than other facial expressions (Ohman et al., 2001). Also for PLDs, an increase in visual sensitivity has been demonstrated for detecting angry walkers (Chouchourelou et al., 2006). Another study showed that the detection of human gait in PLD biological motion correlated with the detection of angry point-light figures, but not happy figures (Ikeda and Watanabe, 2009). It has been proposed that threatening stimuli may be processed more automatically thereby explaining the higher sensitivity to anger in these previous studies. Specifically, the “threat advantage hypothesis” suggests that natural selection resulted in a propensity to react more strongly and automatically to negative than to positive stimuli (Fox et al., 2000). In relation to our study, it can be speculated that tDCS over right pSTS predominantly affected the automatic processing of emotional states, hence the specificity for emotional states with negative emotional valence. However, considering that the present experiment was not *a priori* designed to disentangle the differential effect of stimulation on processing negative/positive–neutral emotional states (limited number of trials for each emotion category), future studies, specifically testing stimulation effects on distinct emotion categories, are warranted to be conclusive on this interpretation.

In our experiment, only the right, not the left pSTS, area was disrupted or stimulated. The suggestion of right hemisphere specialization for processing facial or other non-verbal social cues knows a long history and is based on several clinical observations and neuroimaging studies (Fusar-Poli et al., 2009). Also a very recent study confirmed the notion of right lateralization for perceiving emotional dynamic faces in human pSTS (De Winter et al., 2015). Nevertheless, compensatory contributions of left pSTS or other fronto-parietal regions (e.g., premotor cortex) cannot be ruled out. For example, one study, examining effects of rTMS stimulation over left and right superior temporal lobe, showed that stimulation over both hemispheres can increase attention selectively for angry faces (not for fearful or neutral faces) (Brüne et al., 2006). The latter study therefore indicates that not only right but also left pSTS may play an important role in directing and processing of negative emotional states such as anger. Interestingly, the study by Brüne et al. additionally showed that right hemisphere stimulation more strongly affected processing of male angry faces, whereas right hemisphere stimulation more strongly affected processing of female angry faces. Although preliminary, the authors suggested that this hemispheric specialization might reflect a divergent significance for processing male and female threat (Brüne et al., 2006).

One limitation of this study is the lack of a sham condition (during which no stimulation is applied). Future research is therefore necessary to firmly disentangle whether anodal/cathodal stimulation increased/decreased performance compared to baseline for detecting emotional states with negative emotional valence. Also for the emotion conditions and control task where stimulation showed no effects on performance, a baseline condition is necessary to formally exclude the possibility that anodal and cathodal produced a uniform effect on performance (e.g., both increasing or deteriorating performance). Indeed, in a recent study examining effects of anodal and cathodal tDCS over left primary motor cortex, it was shown that anodal and cathodal tDCS, when applied at similar intensities and duration, can produce similar changes in cortico-motor excitability (Batsikadze et al., 2013).

In summary, the present study found no overall effect of tDCS brain stimulation on bodily emotion recognition from PLDs. However, when emotion categories were explored separately, a significant effect of stimulation was revealed for recognizing emotional states with a negative emotional valence (sadness and anger), as indicated by higher performance during anodal (excitatory) stimulation, compared to cathodal (inhibitory) stimulation over pSTS. This finding partly agrees with previous studies showing structure–function relationships between STS and biological motion processing from PLDs and provides indications that stimulation effects may be modulated by the emotional valence of the stimuli.

## Conflict of Interest Statement

The authors declare that the research was conducted in the absence of any commercial or financial relationships that could be construed as a potential conflict of interest.

## Supplementary Material

The Supplementary Material for this article can be found online at http://journal.frontiersin.org/article/10.3389/fnhum.2015.00438

## References

[B1] Alaerts K. Nackaerts E. Meyns P. Swinnen S. P. Wenderoth N. (2011). Action and emotion recognition from point light displays: an investigation of gender differences. PLoS One 6:6. 10.1371/journal.pone.0020989 21695266 PMC3111458

[B2] Alaerts K. Woolley D. G. Steyaert J. DI M. A. Swinnen S. P. Wenderoth N. (2014). Underconnectivity of the superior temporal sulcus predicts emotion recognition deficits in autism. Soc. Cogn. Affect. Neurosci. 9, 1589–1600. 10.1093/scan/nst156 24078018 PMC4187281

[B3] Allison T. Puce A. Mccarthy G. (2000). Social perception from visual cues: role of the STS region. Trends Cogn. Sci. 4, 267–278. 10.1016/S1364-6613(00)01501-1 10859571

[B4] Arfeller C. Schwarzbach J. Ubaldi S. Ferrari P. Barchiesi G. Cattaneo L. (2013). Whole-brain haemodynamic after-effects of 1-Hz magnetic stimulation of the posterior superior temporal cortex during action observation. Brain Topogr. 26, 278–291. 10.1007/s10548-012-0239-9 22772359

[B5] Atkinson A. P. (2009). Impaired recognition of emotions from body movements is associated with elevated motion coherence thresholds in autism spectrum disorders. Neuropsychologia 47, 3023–3029. 10.1016/j.neuropsychologia.2009.05.019 19500604

[B6] Avenanti A. Annella L. Candidi M. Urgesi C. Aglioti S. M. (2013). Compensatory plasticity in the action observation network: virtual lesions of STS enhance anticipatory simulation of seen actions. Cereb. Cortex 23, 570–580. 10.1093/cercor/bhs040 22426335

[B7] Balconi M. Bortolotti A. (2012). Detection of the facial expression of emotion and self-report measures in empathic situations are influenced by sensorimotor circuit inhibition by low-frequency rTMS. Brain Stimul. 5, 330–336. 10.1016/j.brs.2011.05.004 21782546

[B8] Batsikadze G. Moliadze V. Paulus W. Kuo M. F. Nitsche M. A. (2013). Partially non-linear stimulation intensity-dependent effects of direct current stimulation on motor cortex excitability in humans. J. Physiol. 591(Pt 7), 1987–2000. 10.1113/jphysiol.2012.249730 23339180 PMC3624864

[B9] Boggio P. S. Bermpohl F. Vergara A. O. Muniz A. L. Nahas F. H. Leme P. B. (2007). Go-no-go task performance improvement after anodal transcranial DC stimulation of the left dorsolateral prefrontal cortex in major depression. J. Affect. Disord. 101, 91–98. 10.1016/j.jad.2006.10.026 17166593

[B10] Boggio P. S. Zaghi S. Fregni F. (2009). Modulation of emotions associated with images of human pain using transcranial direct current stimulation (tDCS). Neuropsychologia 47, 212–217. 10.1016/j.neuropsychologia.2008.07.022 18725237

[B11] Brüne M. Bahramali H. Hennessy M. Snyder A. (2006). Are angry male and female faces represented in opposite hemispheres of the female brain? A study using repetitive transcranial magnetic stimulation (rTMS). J. Integr. Neurosci. 5, 187–197. 10.1142/S0219635206001100 16783868

[B12] Candidi M. Stienen B. M. Aglioti S. M. DE Gelder B. (2011). Event-related repetitive transcranial magnetic stimulation of posterior superior temporal sulcus improves the detection of threatening postural changes in human bodies. J. Neurosci. 31, 17547–17554. 10.1523/JNEUROSCI.0697-11.2011 22131416 PMC6623811

[B13] Carrington S. J. Bailey A. J. (2009). Are there theory of mind regions in the brain? A review of the neuroimaging literature. Hum. Brain Mapp. 30, 2313–2335. 10.1002/hbm.20671 19034900 PMC6871093

[B14] Caspers S. Zilles K. Laird A. R. Eickhoff S. B. (2010). ALE meta-analysis of action observation and imitation in the human brain. Neuroimage 50, 1148–1167. 10.1016/j.neuroimage.2009.12.112 20056149 PMC4981639

[B15] Chouchourelou A. Matsuka T. Harber K. Shiffrar M. (2006). The visual analysis of emotional actions. Soc. Neurosci. 1, 63–74. 10.1080/17470910600630599 18633776

[B16] Cutting J. E. Kozlowski L. T. (1977). Recognizing friends by their walk – gait perception without familiarity cues. Bull. Psychon. Soc. 9, 353–356. 10.3758/BF03337021

[B17] De Winter F. L. Zhu Q. Van den Stock J. Nelissen K. Peeters R. de Gelder B. (2015). Lateralization for dynamic facial expressions in human superior temporal sulcus. Neuroimage 106, 340–352. 10.1016/j.neuroimage.2014.11.020 25463458

[B18] di Pellegrino G. Fadiga L. Fogassi L. Gallese V. Rizzolatti G. (1992). Understanding motor events: a neurophysiological study. Exp. Brain Res. 91, 176–180. 10.1007/BF00230027 1301372

[B19] Dittrich W. H. Troscianko T. Lea S. E. G. Morgan D. (1996). Perception of emotion from dynamic point-light displays represented in dance. Perception 25, 727–738. 10.1068/p250727 8888304

[B20] Fadiga L. Fogassi L. Pavesi G. Rizzolatti G. (1995). Motor facilitation during action observation: a magnetic stimulation study. J. Neurophysiol. 73, 2608–2611. 7666169 10.1152/jn.1995.73.6.2608

[B21] Ferrucci R. Giannicola G. Rosa M. Fumagalli M. Boggio P. S. Hallett M. (2012). Cerebellum and processing of negative facial emotions: cerebellar transcranial DC stimulation specifically enhances the emotional recognition of facial anger and sadness. Cogn. Emot. 26, 786–799. 10.1080/02699931.2011.619520 22077643 PMC4234053

[B22] Fox E. Lester V. Russo R. Bowles R. J. Pichler A. Dutton K. (2000). Facial expressions of emotion: are angry faces detected more efficiently? Cogn. Emot. 14, 61–92. 10.1080/026999300378996 17401453 PMC1839771

[B23] Freitag C. M. Konrad C. Haberlen M. Kleser C. Von G. A. Reith W. (1994). Perception of biological motion in autism spectrum disorders. Neuropsychologia 46, 1480–1494. 10.1016/j.neuropsychologia.2007.12.025 18262208

[B24] Fusar-Poli P. Placentino A. Carletti F. Landi P. Allen P. Surguladze S. (2009). Functional atlas of emotional faces processing: a voxel-based meta-analysis of 105 functional magnetic resonance imaging studies. J. Psychiatry Neurosci. 34, 418–432. 19949718 PMC2783433

[B25] Gallese V. Keysers C. Rizzolatti G. (2004). A unifying view of the basis of social cognition. Trends Cogn. Sci. 8, 396–403. 10.1016/j.tics.2004.07.002 15350240

[B26] Grafton S. T. Arbib M. A. Fadiga L. Rizzolatti G. (1996). Localization of grasp representations in humans by positron emission tomography.2. Observation compared with imagination. Exp. Brain Res. 112, 103–111. 10.1007/BF00227183 8951412

[B27] Grossman E. D. Battelli L. Pascual-Leone A. (2005). Repetitive TMS over posterior STS disrupts perception of biological motion. Vision Res. 45, 2847–2853. 10.1016/j.visres.2005.05.027 16039692

[B28] Hein G. Knight R. T. (2008). Superior temporal sulcus – it’s my area: or is it? J. Cogn. Neurosci. 20, 2125–2136. 10.1162/jocn.2008.20148 18457502

[B29] Hubert B. Wicker B. Moore D. G. Monfardini E. Duverger H. Da Fonseca D. (2007). Brief report: recognition of emotional and non-emotional biological motion in individuals with autistic spectrum disorders. J. Autism Dev. Disord. 37, 1386–1392. 10.1007/s10803-006-0275-y 17160459

[B30] Iacoboni M. Woods R. P. Brass M. Bekkering H. Mazziotta J. C. Rizzolatti G. (1999). Cortical mechanisms of human imitation. Science 286, 2526–2528. 10.1126/science.286.5449.2526 10617472

[B31] Ikeda H. Watanabe K. (2009). Anger and happiness are linked differently to the explicit detection of biological motion. Perception 38, 1002–1011. 10.1068/p6250 19764302

[B32] Jastorff J. Orban G. A. (2009). Human functional magnetic resonance imaging reveals separation and integration of shape and motion cues in biological motion processing. J. Neurosci. 29, 7315–7329. 10.1523/JNEUROSCI.4870-08.2009 19494153 PMC6666481

[B33] Johansson G. (1973). Visual-perception of biological motion and a model for its analysis. Percept. Psychophys. 14, 201–211. 10.3758/BF0321237815820512

[B34] Kaiser M. D. Pelphrey K. A. (2012). Disrupted action perception in autism: behavioral evidence, neuroendophenotypes, and diagnostic utility. Dev. Cogn. Neurosci. 2, 25–35. 10.1016/j.dcn.2011.05.005 22682727 PMC6987680

[B35] Maeoka H. Matsuo A. Hiyamizu M. Morioka S. Ando H. (2012). Influence of transcranial direct current stimulation of the (dorsolateral) prefrontal cortex on pain related emotions: a study using electroencephalographic power spectrum analysis. Neurosci. Lett. 512, 12–16. 10.1016/j.neulet.2012.01.037 22326385

[B36] Molenberghs P. Brander C. Mattingley J. B. Cunnington R. (2010). The role of the superior temporal sulcus and the mirror neuron system in imitation. Hum. Brain Mapp. 31, 1316–1326. 10.1002/hbm.20938 20087840 PMC6870593

[B37] Molenberghs P. Cunnington R. Mattingley J. B. (2012). Brain regions with mirror properties: a meta-analysis of 125 human fMRI studies. Neurosci. Biobehav. Rev. 36, 341–349. 10.1016/j.neubiorev.2011.07.004 21782846

[B38] Nackaerts E. Wagemans J. Helsen W. Swinnen S. P. Wenderoth N. Alaerts K. (2012). Recognizing biological motion and emotions from point-light displays in autism spectrum disorders. PLoS ONE 7:9. 10.1371/journal.pone.0044473 22970227 PMC3435310

[B39] Nitsche M. A. Koschack J. Pohlers H. Hullemann S. Paulus W. Happe S. (2012). Effects of frontal transcranial direct current stimulation on emotional state and processing in healthy humans. Front. Psychiatry 3:58. 10.3389/fpsyt.2012.00058 22723786 PMC3377009

[B40] Nitsche M. A. Paulus W. (2000). Excitability changes induced in the human motor cortex by weak transcranial direct current stimulation. J. Physiol. 527, 633–639. 10.1111/j.1469-7793.2000.t01-1-00633.x 10990547 PMC2270099

[B41] Ohman A. Lundqvist D. Esteves F. (2001). The face in the crowd revisited: a threat advantage with schematic stimuli. J. Pers. Soc. Psychol. 80, 381–396. 10.1037/0022-3514.80.3.381 11300573

[B42] Parron C. da Fonseca D. Santos A. Moore D. G. Monfardini E. Deruelle C. (2008). Recognition of biological motion in children with autistic spectrum disorders. Autism 12, 261–274. 10.1177/1362361307089520 18445735

[B43] Pelphrey K. A. Shultz S. Hudac C. M. Wyk B. C. V. (2011). Research review: constraining heterogeneity: the social brain and its development in autism spectrum disorder. J. Child Psychol. Psychiatry 52, 631–644. 10.1111/j.1469-7610.2010.02349.x 21244421 PMC3096715

[B44] Pitcher D. (2014). Facial expression recognition takes longer in the posterior superior temporal sulcus than in the occipital face area. J. Neurosci. 34, 9173–9177. 10.1523/JNEUROSCI.5038-13.2014 24990937 PMC6608256

[B45] Plazier M. Joos K. Vanneste S. Ost J. de Ridder D. (2012). Bifrontal and bioccipital transcranial direct current stimulation (tDCS) does not induce mood changes in healthy volunteers: a placebo controlled study. Brain Stimulat. 5, 454–461. 10.1016/j.brs.2011.07.005 21962976

[B46] Pollick F. E. Kay J. W. Heim K. Stringer R. (2005). Gender recognition from point-light walkers. J. Exp. Psychol. Hum. Percept. Perform. 31, 1247–1265. 10.1037/0096-1523.31.6.1247 16366787

[B47] Pollick F. E. Lestou V. Ryu J. Cho S. B. (2002). Estimating the efficiency of recognizing gender and affect from biological motion. Vision Res. 42, 2345–2355. 10.1016/S0042-6989(02)00196-7 12350423

[B48] Redcay E. (2008). The superior temporal sulcus performs a common function for social and speech perception: implications for the emergence of autism. Neurosci. Biobehav. Rev. 32, 123–142. 10.1016/j.neubiorev.2007.06.004 17706781

[B49] Rizzolatti G. Craighero L. (2004). The mirror-neuron system. Annu. Rev. Neurosci. 27, 169–192. 10.1146/annurev.neuro.27.070203.144230 15217330

[B50] Rizzolatti G. Fabbri-Destro M. (2008). The mirror system and its role in social cognition. Curr. Opin. Neurobiol. 18, 179–184. 10.1016/j.conb.2008.08.001 18706501

[B51] Saygin A. P. (2007). Superior temporal and premotor brain areas necessary for biological motion perception. Brain 130, 2452–2461. 10.1093/brain/awm162 17660183

[B52] van Kemenade B. M. Muggleton N. Walsh V. Saygin A. P. (2012). Effects of TMS over premotor and superior temporal cortices on biological motion perception. J. Cogn. Neurosci. 24, 896–904. 10.1162/jocn_a_00194 22264195

[B53] Zhang X. Woolley D. G. Swinnen S. P. Feys H. Meesen R. Wenderoth N. (2014). Changes in corticomotor excitability and intracortical inhibition of the primary motor cortex forearm area induced by anodal tDCS. PLoS ONE 9:e101496. 10.1371/journal.pone.0101496 24999827 PMC4084808

