# Supplementary material for: Effects of Transcranial Direct Current Stimulation on the Recognition of Bodily Emotions from Point-Light Displays
Source: Front Hum Neurosci. 2015 Aug 3;9:438. doi: 10.3389/fnhum.2015.00438 (PMC4522557; doi:10.3389/fnhum.2015.00438)

Does transcranial direct-current stimulation over the superior temporal sulcus enhance the recognition of emotions from point light displays?

Authors

Sharona Vonck, Stephan P. Swinnen, Nicole Wenderoth, Kaat Alaerts

Supplementary Figure 1.

Effects of tDCS over right STS

Panel A visualizes **accuracy scores** on the emotion and control task while participants received anodal (excitatory) (black circles) or cathodal (inhibitory) tDCS (grey squares).

Panel B visualizes **reaction times** on the emotion and control task while participants received anodal (excitatory) (black circles) or cathodal (inhibitory) tDCS (grey squares).

Effects of stimulation are visualized separately for each emotional state (neutral, happy, sad, angry). Vertical bars denote standard errors.

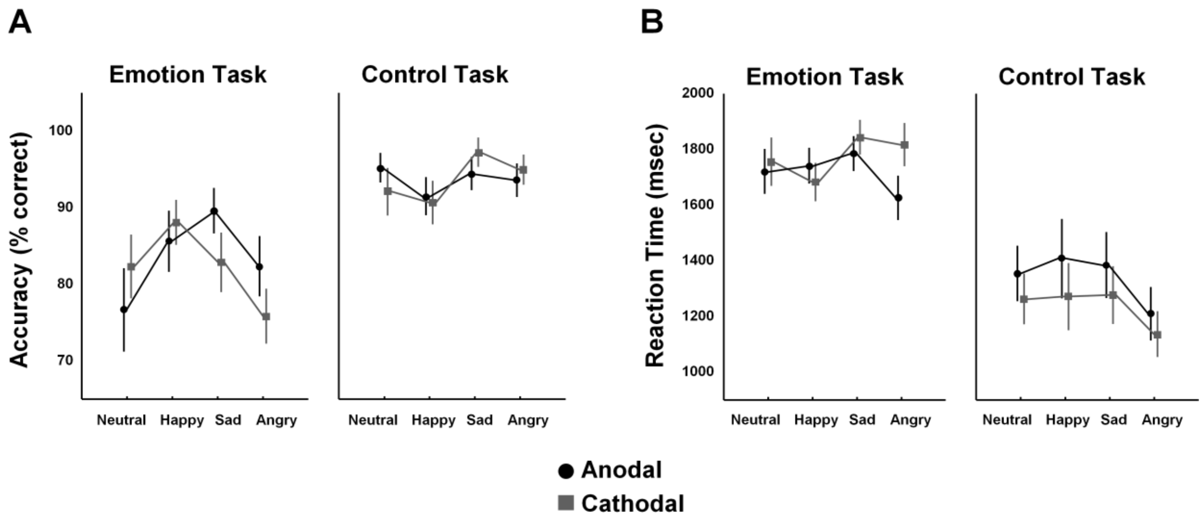

Supplement: Supplementary file 1 [file Image_1.PDF]
